# L-looped Transposition of the Great Arteries in a Patient with Marfanoid Habitus: First Reported Case in Literature

**DOI:** 10.7759/cureus.5416

**Published:** 2019-08-18

**Authors:** Abdul Majid, Syed Hamza Bin Waqar, Farah Yasmin, Osama Mohiuddin, Anosh Aslam Khan

**Affiliations:** 1 Cardiology, Civil Hospital Karachi, Dow University of Health Sciences, Karachi, PAK; 2 Internal Medicine, Civil Hospital Karachi, Dow University of Health Sciences, Karachi, PAK; 3 Internal Medicine, Dow University of Health Sciences, Karachi, PAK

**Keywords:** transposition of great arteries, vsd, pda, marfanoid, discordancy, l-tga, transposition of great arteries, dextrocardia, atrial-switch

## Abstract

L-looped transposition of great arteries (L-TGA) is an extremely rare heart condition. It is associated with physiologically corrected transposition of great vessels, leading to the normal return of deoxygenated systemic venous blood to the heart and transport of oxygenated pulmonary venous blood to the main systemic circulation. Anatomic discordancy and anomalous coronary artery distribution predispose the right ventricle to excessive workload and eventual heart failure. This mandates anatomic correction or proper medical management of heart failure. Herein, we present a case of a 14-year-old girl who presented to our cardiology consults with a false impression of pre-made clinical diagnosis of tetralogy of Fallot. She had increasing dyspnea and cyanosis on presentation. Striking marfanoid habitus and unusual echocardiographic findings of tripartite geometry of heart with parallel and discordant positioning of atria, ventricles, and great arteries led us to cardiac computed tomography which confirmed the diagnosis of L-TGA. Our patient also had associated patent ductus arteriosus, dextrocardia, ventricular septal defect (VSD), and pulmonary atresia. Due to the complex nature of heart disease and unavailability of resources, she was treated with a comprehensive heart failure protocol and followed up clinically and radiologically at regular intervals and showed massive improvement. This is the first-ever documented case of L-TGA with complex shunting and marfanoid habitus.

## Introduction

L-transposition of great arteries (L-TGA), also known as congenitally corrected transposition of great arteries (ccTGA), is an extremely rare clinical manifestation of cyanotic heart diseases. It mostly remains hidden throughout childhood secondary to the physiological shunting through the anatomically discordant atrioventricular and ventriculoarterial connections [1]. The incompetent right ventricle is faced with an enormous workload and eventual heart failure is the ultimate destiny in patients with L-TGA if it remains undiagnosed and untreated [2]. Herein, we present the first case of its kind in literature which contrasts the presence of marfanoid habitus with complex shunts with L-TGA. Our patient presents in young age with signs of heart failure and was managed non-surgically on neurohormonal blockade principles of heart failure management and showed massive improvement in hemodynamic parameters, growth, and symptomatology.

## Case presentation

A 14-year-old female patient, previously diagnosed as a case of cyanotic heart disease since birth, presented to our cardiology department with the complaint of increasing shortness of breath (SOB) for the past two days. According to patient’s attendant, she had a bluish discoloration of fingers, toes, and tongue since childhood, which aggravated while crying and whenever the child got ill. She was later clinically diagnosed under the false impression of teratology of fallot and was managed conservatively. Moreover, for the last four years, she complained of increasing SOB that limited her activities of daily living. Months preceding the presentation, SOB progressed from climbing two steps of stairs to difficulty in catching a breath at rest for which she was prescribed loop diuretics which only partially resolved her symptomatology. She had no history of orthopnea, joint pain, paroxysmal nocturnal dyspnea, chest pain, cough, rash, and fever. She also complained of hematemesis which was coffee ground color, two to three episodes twice in the year preceding the presentation. It was accompanied with complains of having black stools on and off for which endoscopy was advised but the patient refused it. Her family history was insignificant for any cardiac structural heart or collagen vascular disease.

**Figure 1:**
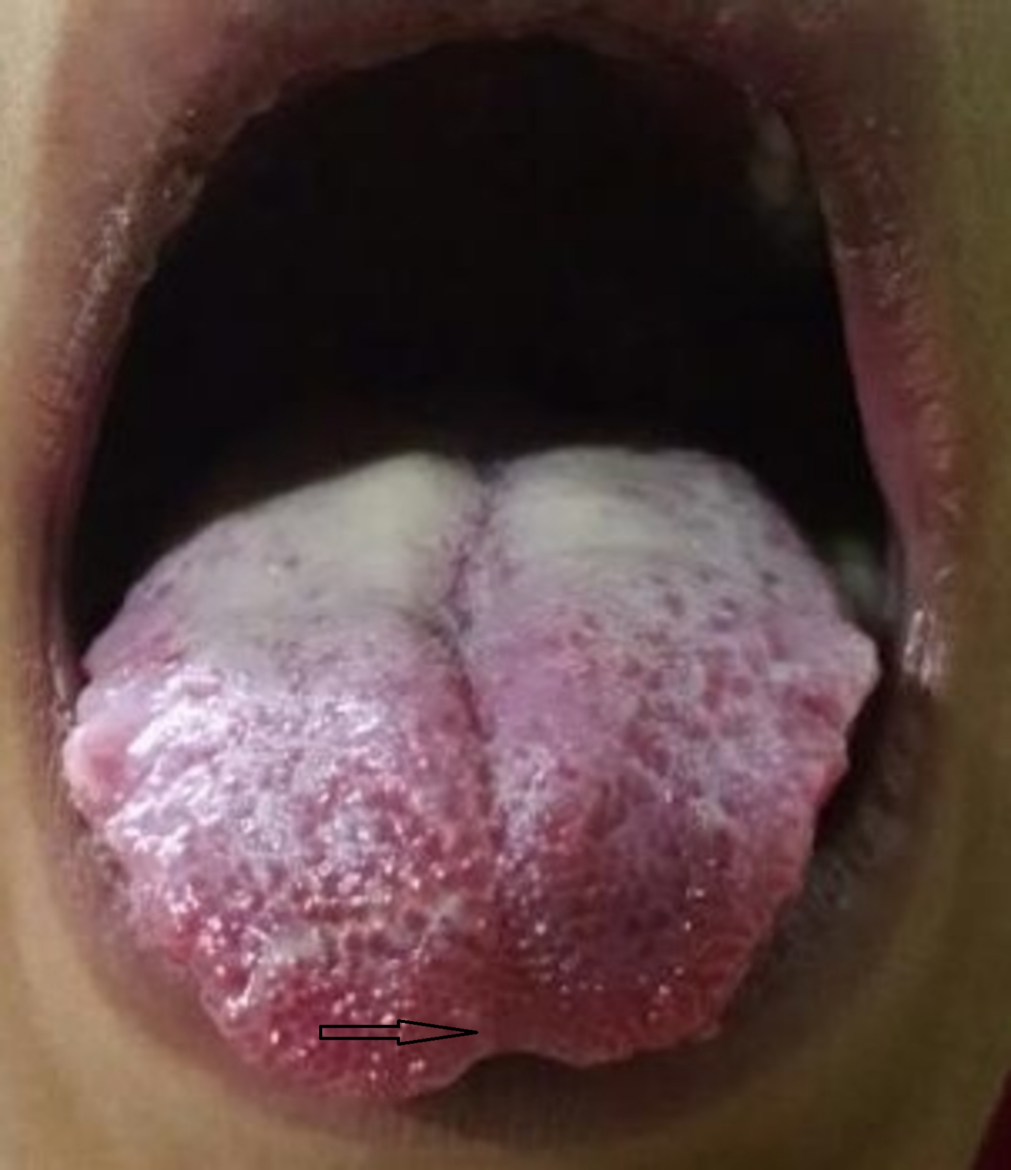
BIfid tongue (indicated by the arrow) on examination in a patient with marfanoid features

**Figure 2:**
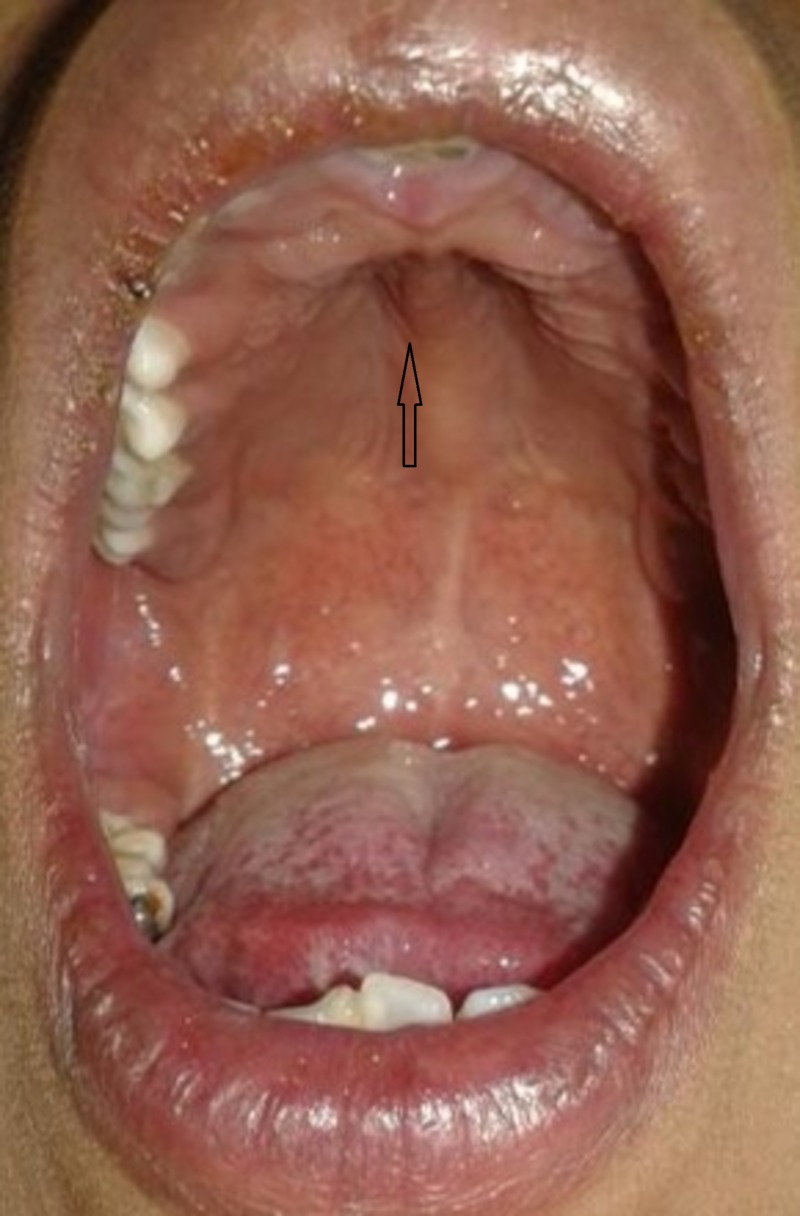
High-arched palate (indicated by an arrow)

**Figure 3:**
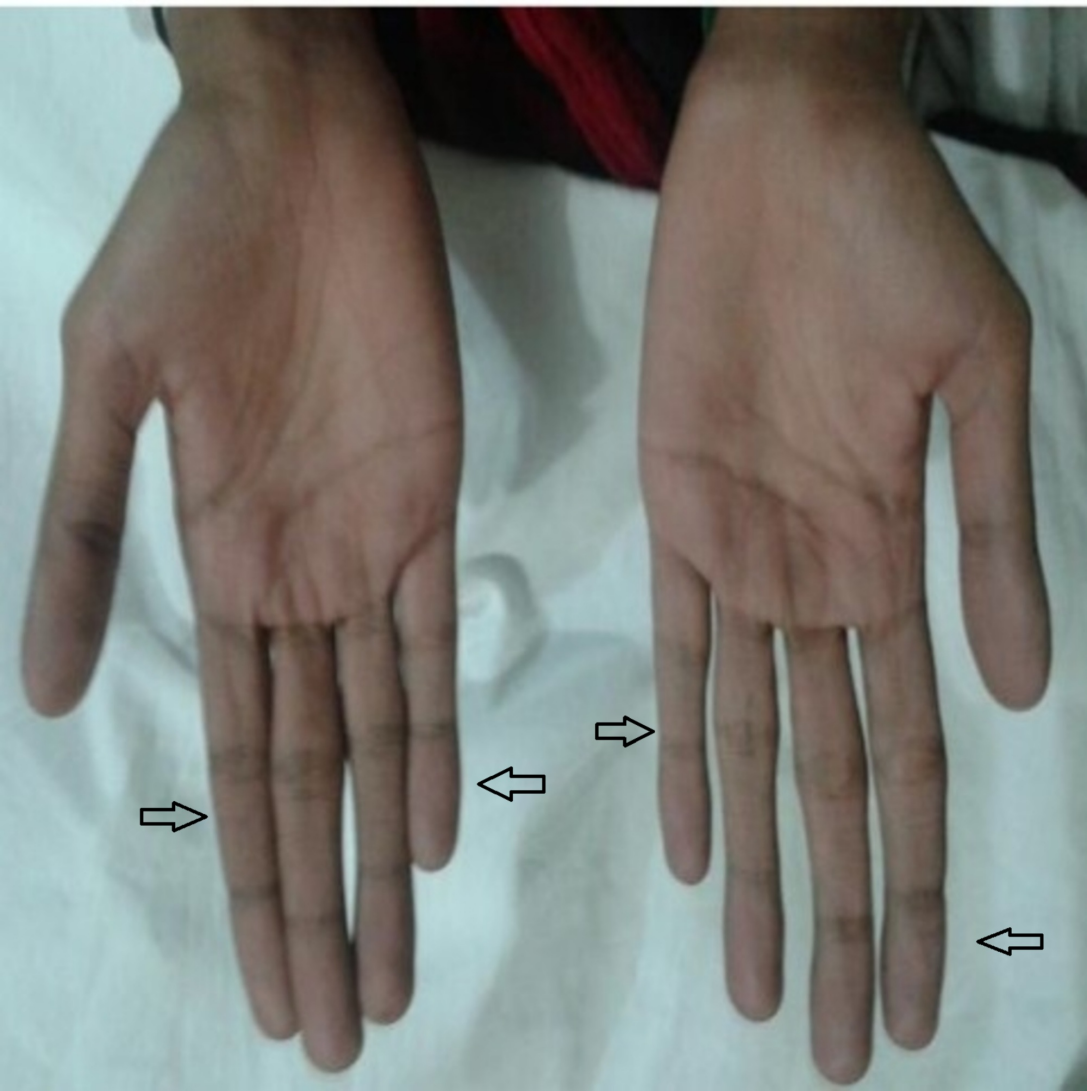
Thin, slender and long fingers (indicated by the arrows) consistent with marfanoid features

**Figure 4:**
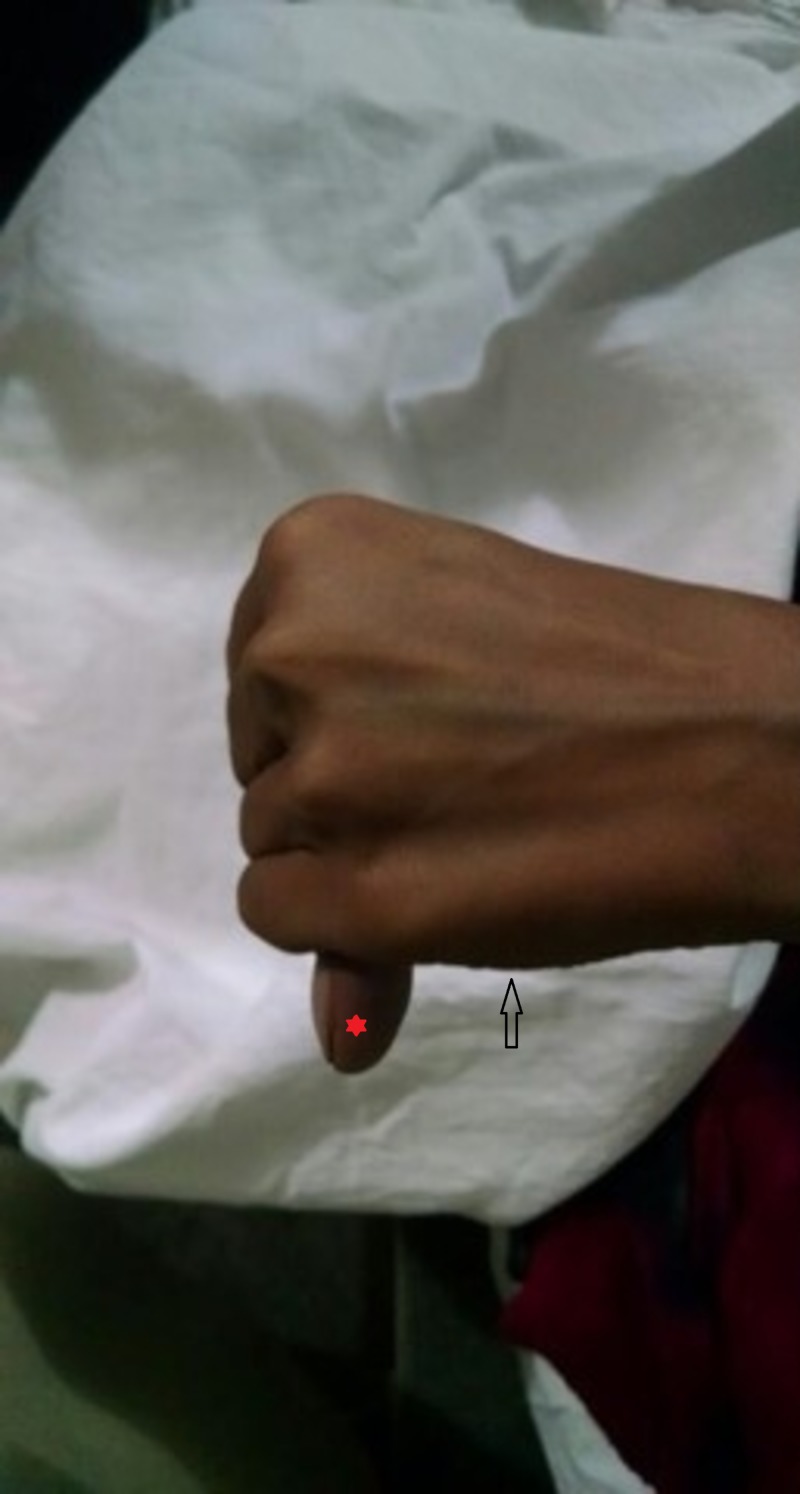
Positive Steinberg sign The entire distal phalanx of the thumb (*) extends beyond the ulnar border of the clenched fist (indicated by the arrow).

**Figure 5:**
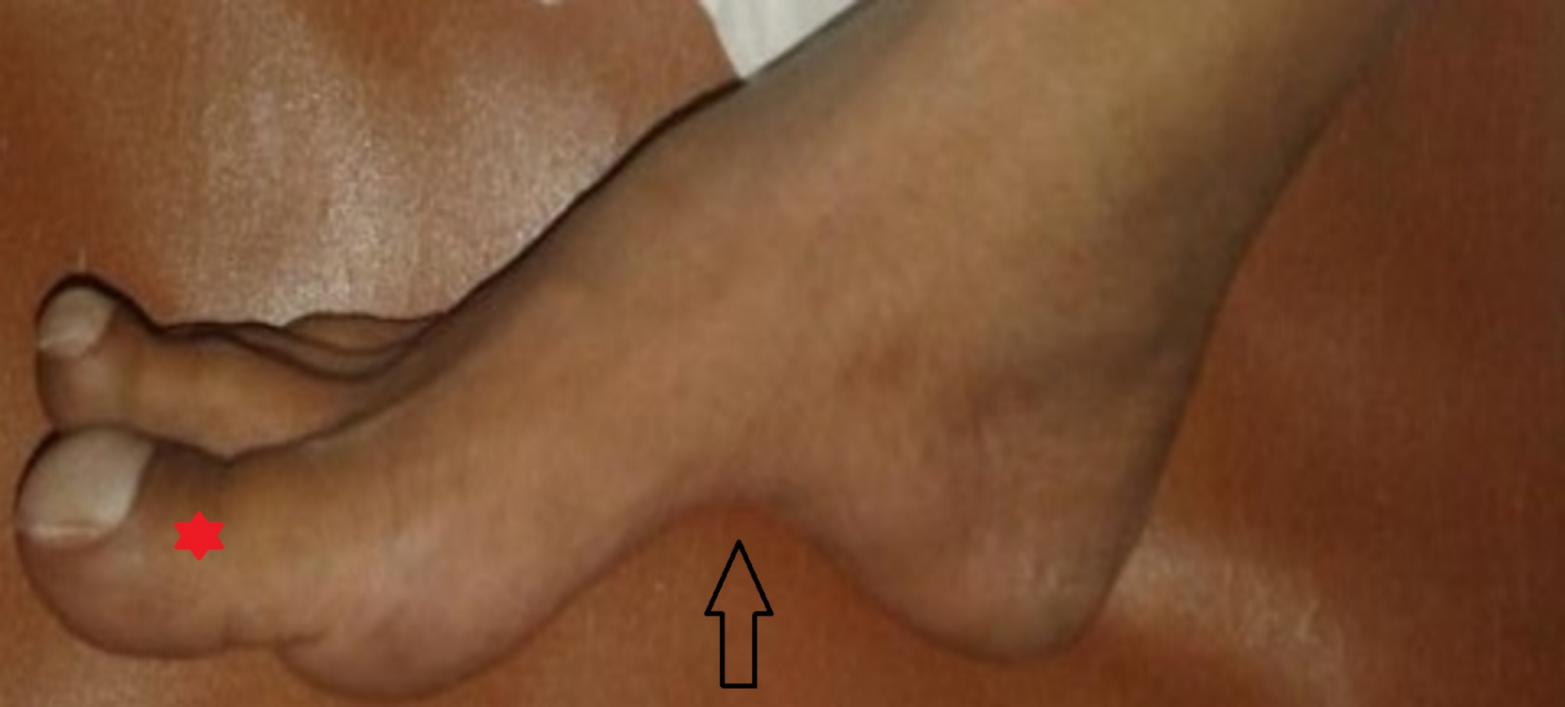
Prominent pes cavus (shown with arrow) with drum-stick appearance of toes (*) consistent with cyanotic heart disease

On admission, she looked frail and cyanotic with visible discomfort. She was oriented to time, place and person. The patient was afebrile with a pulse rate of 100 beats per minute (BPM), blood pressure (BP) of 100/60 mmHg, and a respiratory rate of 24 per minute with an oxygen saturation of 60%. The patient had dental carries, thin and bifid cyanosed tongue with a high arched palate, arm span more than height, thin long slender fingers, positive Steinberg sign, and pes cavus [Figures 1-5].

These findings were highly suggestive of marfanoid habitus and were discordant to the cardiorespiratory findings of our patient as most marfanoid patients present with pathologies inflicting the aortic vessel. The cardiovascular exam showed apex beat which was palpable at fifth intercostal space, one centimeter medial to the midclavicular line, and normal in character. The cardiac rhythm was regular with a grade three systolic murmur audible along the lower right sternal border that increases with inspiration. A continuous murmur was also heard over the right infraclavicular area. A single S2 was also heard at the second right intercostal space. No added heart sounds were noted. The pulmonary exam did not yield any abnormal findings. The spirometry was also normal and showed no obstructive or restrictive lung pattern. The neurological exam for bulk, tone, power, and reflexes was insignificant for any finding. Her abdomen was soft, non-tender, non-distended with normoactive bowel sounds and evidence of slight hepatomegaly. The patient had significant clubbing with central and peripheral cyanosis. There were no signs of scleral icterus, dehydration, edema, puffiness or conjunctival pallor.

The complete blood count (CBC) showed a hemoglobin (Hb) of 16 mg/dl and a total leukocyte count (TLC) of 6.5 x 109/L with neutrophils being 68% and lymphocytes being 10%. The mean corpuscular volume (MCV) of 67.5 femtolitres (fL) with a hematocrit of 64% and platelets of 430 x 103 per cubic millimeters was noted. The basic metabolic panel indicated normal sodium, potassium, and chloride levels. The blood urea nitrogen (BUN) and creatinine were also within normal limits. Uric acid levels were marginally elevated.

The liver function tests (LFT) were within normal limits. The coagulation profile was deranged with an INR of 5.4 for which mandated six-packs of fresh frozen plasma transfusion.

Chest X-rays (CXR), electrocardiography (ECG), echocardiography (ECHO), and ultrasound (US) abdomen were carried out for further diagnosis.

**Figure 6:**
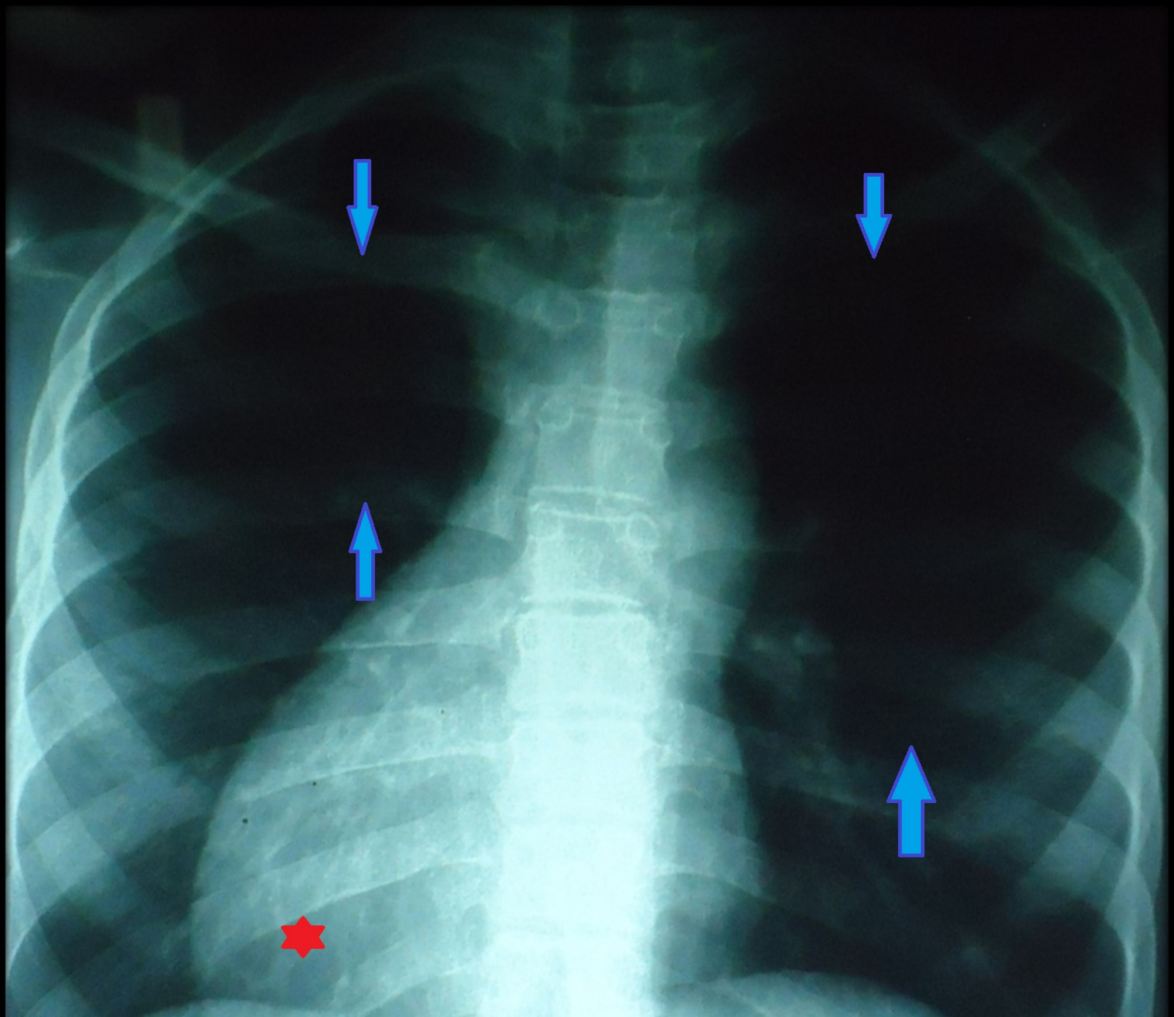
Chest X-ray PA view showing oligemic lung fields (shown in between the arrows) and dextrocardia (*) shown as inverted position of apex of heart towards the right side PA, posteroanterior

The CXR showed moderate right ventricular enlargement with dextrocardia with oligemic lung fields (Figure 6).

The ECG showed changes in right precordial leads with some straining and right axis deviation. There were septal Q waves in right precordial leads (QR pattern in V4R and V1 leads) and absent Q waves in some left precordial leads.

The ultrasound abdomen was significant for mild enlargement of the liver with possible coarse echotexture of hepatic parenchyma secondary to iron overload.

**Figure 7:**
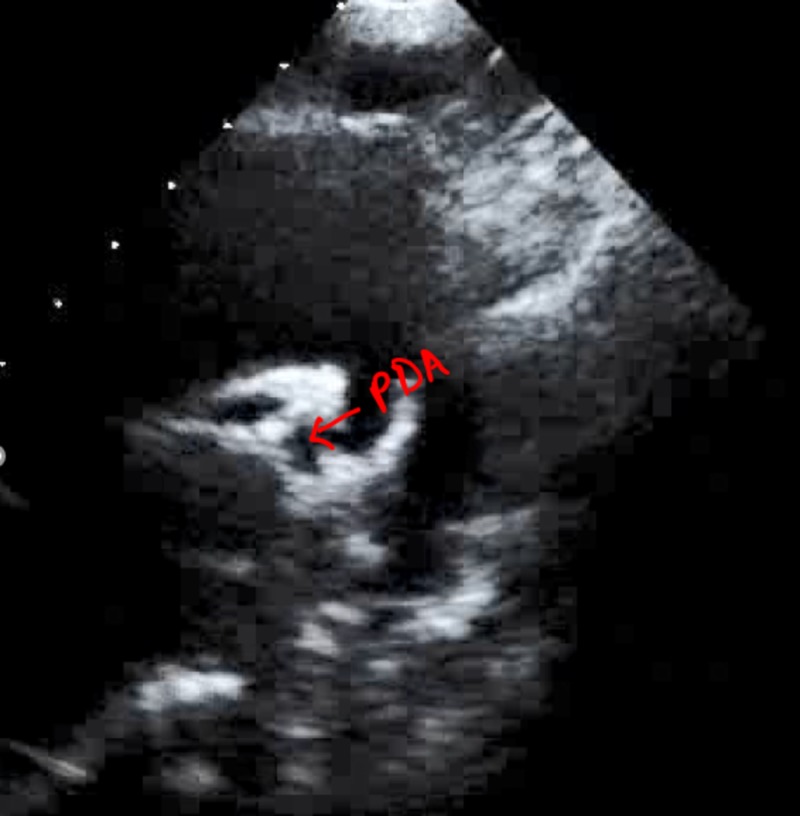
ECHO showing PDA ECHO, echocardiography; PDA, patent ductus arteriosus

The ECHO study of the heart was done to investigate the atypical clinical and radiological findings. It showed complex shunting of heart chambers and arteries with discordance between atria, ventricles and great arteries. It showed the presence of systemic position of tricuspid valve with no apical displacement and morphologic right ventricle. The tricuspid valve was seen as inferior to the mitral valve. Great arteries of the heart were running parallel and clear looping of the ventricles with the septal defect was seen. Ejection fraction was noted to be around 60% (Figure 7).

**Figure 8:**
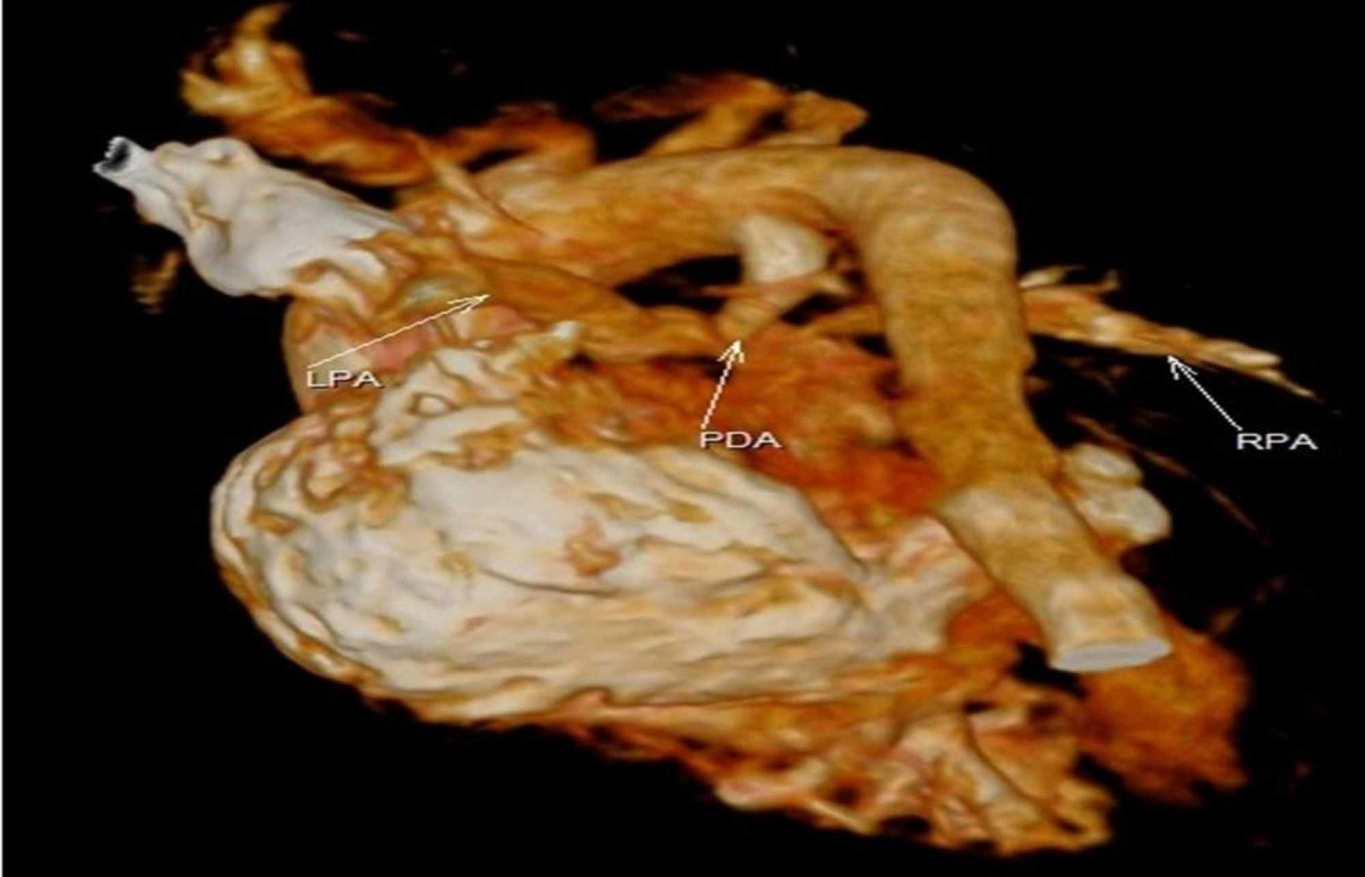
Cardiac volume computed tomography showing anomalous positioning of LPA with PDA and aortic discordance RPA, right pulmonary artery; LPA, left pulmonary artery; PDA, patent ductus arteriosus

**Figure 9:**
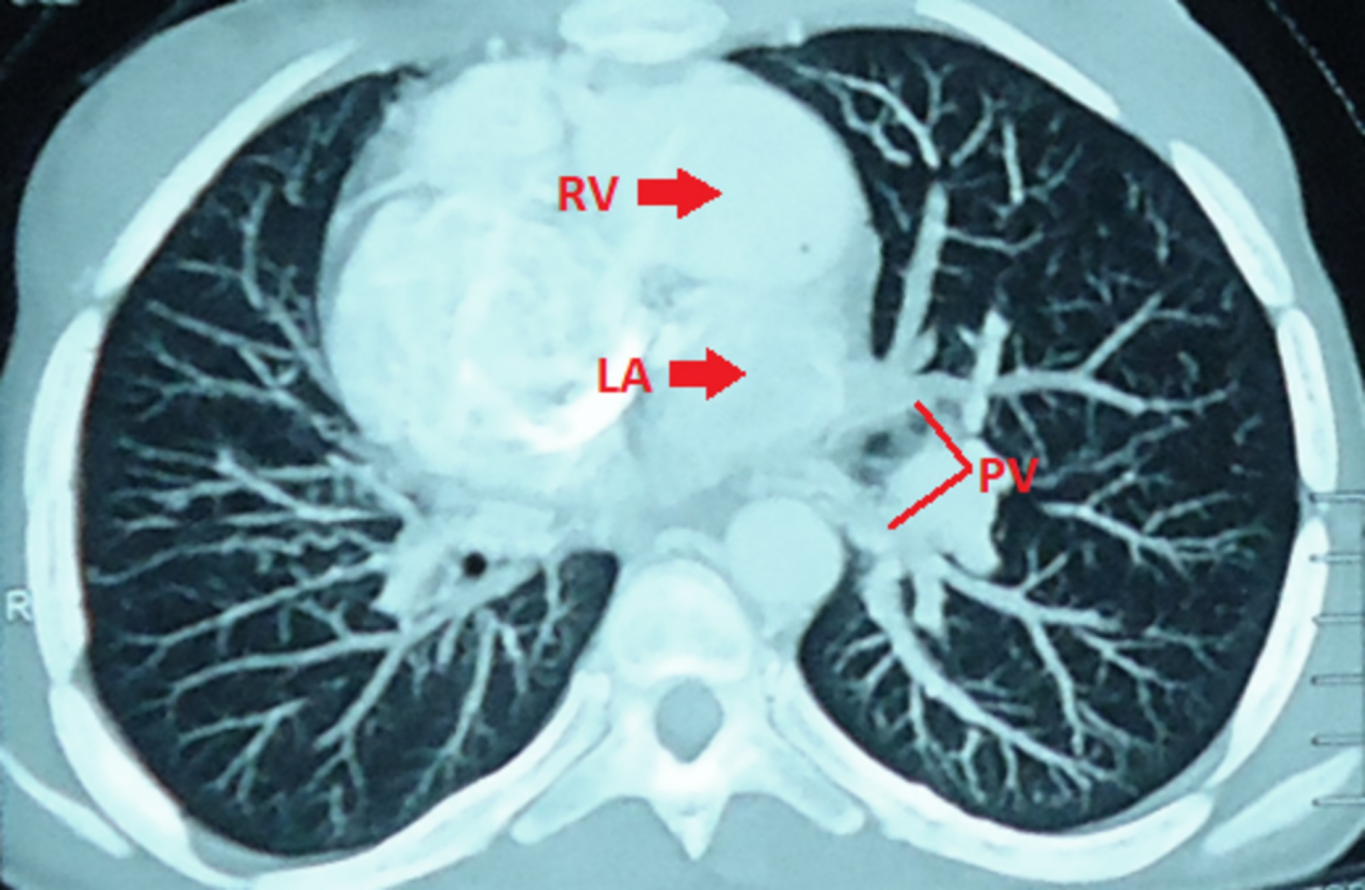
Cardiac computed tomography showing discordant positioning of LA and RV PV, Pulmonary vein; LA, left atrium; RV, right ventricle

**Figure 10:**
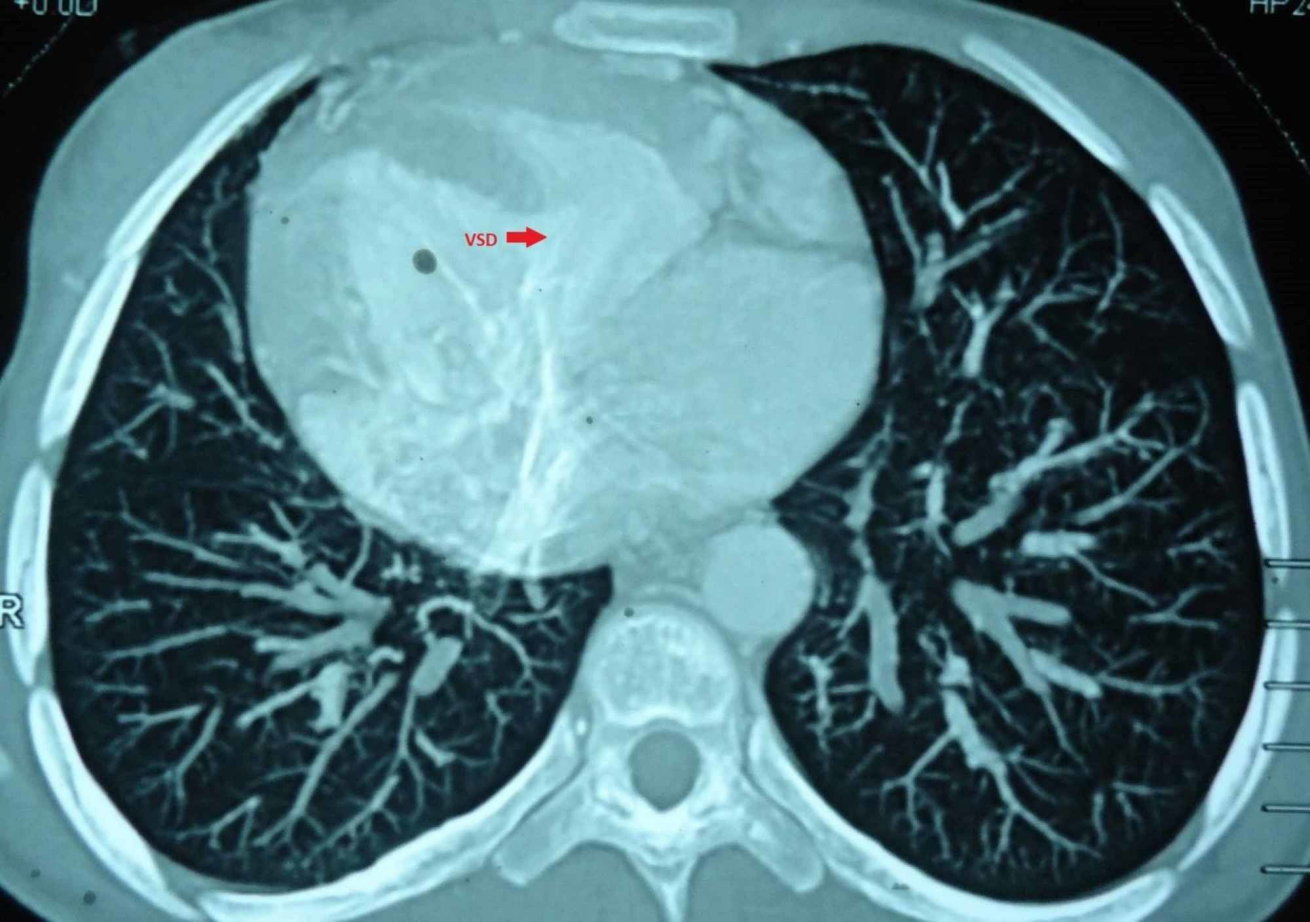
Cardiac computed tomography showing huge VSD VSD, ventricular septal defect

Cardiac computed tomography (CT) was carried out for further evaluation. It showed the ventricular septal defect (VSD), patent ductus arteriosus, pulmonary atresia, dextrocardia and transposition of great arteries (Figures 8-10).

Cardiac CT confirmed the unusual presentation of ccTGA.

A multi-disciplinary team was set up and the collective decision of conservative treatment was concluded for our patient. She was started on oxygen support, cautious intravenous saline solution, spironolactone, propranolol, and loop diuretics to stabilize the hemodynamic parameters. Two sets of phlebotomies were also conducted with iron replacement with ferrous gluconate. Cardiac surgery was withheld due to unavailability of possible resources to reverse complex cardiac shunting and impressive control of hemodynamic parameters on optimal medical management. She was followed up weekly for three weeks and later, monthly for consecutive six months. Follow-up revealed marked improvement in SOB and saturation levels. Proper vaccination for her condition and nutritional supplementation were also done to achieve better results.

## Discussion

ccTGA, also known as L-transposition, presents with a multitude of clinical presentations depending upon the presence and severity of associated cardiac lesions and the development of right ventricular (RV) dysfunction [1]. The outcomes of a pooled analysis showed ccTGA to be associated with atrial fibrillation and anomalies like inverse coronary artery and coronary stenosis, progressive tricuspid regurgitation, pulmonary stenosis, and ventricular septal defects (VSD). The latter three cardiac lesions are present in over 90% of the patients due to morphologic RV and delicate tricuspid valve supporting the systemic circulation as a result of both discordant atrioventricular and ventriculoarterial connections. Furthermore, the anomalous location of the atrioventricular (AV) node along with the AV bundle leads to an abnormal cardiac conduction system and hence arrhythmias in these patients [2]. The presence of a left ventricular outflow tract obstruction, VSD, and pulmonary atresia leads to cyanosis due to decreased pulmonary blood flow, increased pulmonary vascular resistance, and right to left shunting of blood [3].

However, our patient also presented with dextrocardia which is a relatively rare anatomic variant of ccTGA with similar pathophysiology, accounting for only two out of the twenty-two cases of a case series conducted in New Zealand [4]. A multi-centered study conducted by Graham et al., comprising a cohort of 182 L-TGA patients illustrated two-thirds (67%) of the sample with associated cardiac anomalies and one third (25%) without any related lesions to develop congestive heart failure (CHF) by the age of 45 years [5]. Tricuspid regurgitation is a major risk factor for CHF due to increased volume and pressure overload on the morphologic RV acting as the systemic pump [6]. However, patients presenting with associated VSD and pulmonary stenosis are protected against CHF, resulting in better outcomes due to increased afterload on left ventricle reducing the interventricular septal shift to the LV resulting in less deterioration of RV function and delays the tricuspid regurgitation [7].

Moreover, long-standing polycythemia leading to increased uric acid production due to cyanotic congenital heart diseases like L-TGA is not commonly recognized. Cyanosis over a period of more than 10 years, higher Hb levels, and polycythemia are some of the factors contributing to the development of hyperuricemia [8].

Looped TGA is usually diagnosed based on its characteristic discordant findings in electrocardiography (ECG) showing Q waves in the right-sided precordial leads and absence of Q waves in the left-sided precordial leads due to interventricular septum being depolarized in the opposite direction. This can often be misinterpreted as inferior myocardial infarction [2,9]. Furthermore, the associated cardiac lesions are evaluated using Echocardiography (ECHO).

L-TGA is diagnosed by the (S, L, L) presentation representing atrial situs solitus, i.e., normal positioning of atrium (right-sided inferior and superior vena cavae returning deoxygenated blood to a right-sided pulmonary atrium), L-looped ventricles with the systemic location of the tricuspid valve and morphologic right ventricle on the patient’s left, while the morphologic left ventricle and mitral valve on the right and L-transposed great arteries with the aorta arising from the left-sided morphologic right ventricle and hence anterior, superior and to the left of pulmonary artery along with parallel positioning of the great arteries [10].

Ventricular morphology can be differentiated from the short axis and apical four-chamber views with the right ventricle characterized by a coarse and left ventricle with fine trabeculations. Chest radiograph may be performed to evaluate the mesocardia (apex of heart located in midline of thorax) and dextrocardia (apex of heart pointing to the right) accounting for approximately 20% of these cases as compared to levocardia (normally located i.e. apex to the left) but the leftward positioned aorta producing a prominence in the upper left border of mediastinum [2]. VSD can be found in any region of the interventricular septum, large, peri-membranous, and subpulmonary in the location, being the most common. Muscular- trabecular defects and multiple VSDs can also be seen [10].

The mainstay of medical treatment for ccTGA patients is to reduce the afterload on the RV by decreasing the systemic pressure on its wall and slow remodeling to improve RV dysfunction. The patient is usually prescribed angiotensin-converting enzyme inhibitors (ACE-I), angiotensin receptor blockers (ARBs) and β-blockers. Digoxin may also be prescribed for its anti-arrhythmic effects. Furthermore, the surgical approach is usually decided based on the age of presentation and the extent of the cardiac lesions. In patients with only VSD with no left pulmonary outflow tract obstruction, ‘’classic’’ or ‘’physiologic’’ repair involving a VSD patch suture is employed to close the VSD defect only. Another surgical approach, known as the “anatomic” or “Double Switch” (DS) procedure is employed in case of unsatisfactory outcomes from the classic repair. This approach includes the closure of VSD, coronary artery transfer and arterial switch along with interatrial baffle by Senning or Mustard procedure. The Senning and Mustard procedure, referred to as an “atrial switch,” directs the systemic venous flow to the tricuspid valve and right ventricle and pulmonary venous flow to the mitral valve and left ventricle , thus improving the long term outcome by restoring the left ventricular and mitral valve to the systemic circulation [10]. However, the suitable surgical procedure for most of TGA cases in children with VSD is an arterial switch operation [11]. There have been multiple case reports on patients of older age who are asymptomatic and hence undiagnosed until the fourth and fifth decades of life [12-13]. However, this study presents the case of a young 14-year-old female showing progressive SOB due to multiple complex cardiac defects from a developing country like Pakistan.

## Conclusions

L-TGA can unusually present symptomatically at a young age as in our case. In such presentations, every effort should be made to find multiple shunts and aggressive medical therapy should be started. Surgical correction should be opted, wherever possible. This is the first case of its nature in literature contrasting marfanoid habitus with discordance of major arteries and ventricles. Further case reports and studies are needed to ascertain the pathophysiology and research should be conducted to find the underlying etiopathogenesis and possible genetic defects.
